# Targeted Therapy in Ewing Sarcoma

**DOI:** 10.5402/2012/609439

**Published:** 2012-05-28

**Authors:** A. Lissat, M. M. Chao, U. Kontny

**Affiliations:** ^1^Division of Pediatric Hematology and Oncology, Department of Pediatrics, University Medical Center Freiburg, 79106 Freiburg, Germany; ^2^Division of Pediatric Hematology and Oncology, Children's National Medical Center, Washington, DC 20010, USA

## Abstract

Despite marked improvement in the prognosis of patients with nonmetastatic Ewing sarcoma (ES), the outcome for patients with recurrent or metastatic disease remains poor. Insight into key biologic processes in ES could provide new therapeutic targets. The particular biologic feature of ES, the fusion of the *EWS* gene with a member of the *ETS* family of genes, is present in >95% of cases. The EWS-ETS chimeric protein leads to aberrant transcription that promotes tumor initiation and propagation via prosurvival and antiapoptotic pathways. Recent research has identified cooperating mutations important for ES tumorigenesis. This paper provides a summary of the latest research in ES and discusses potential novel targets for therapy.

## 1. Introduction

Ewing sarcoma is the second most common malignant bone tumor in children and adolescents. The tumor consists of small blue round cells and is characterized by a translocation between *EWS* and a member of the *ETS* transcription factor family. The translocation is also found in Askin's tumor, extraosseous Ewing sarcoma (EES), and peripheral primitive neuroectodermal tumors (pPNETs) which together with Ewing sarcoma comprise the Ewing sarcoma family of tumors (EFST), in the following referred as Ewing sarcoma (ES). The annual incidence of ES is three in 1 million children under 15 years with 30% of patients presenting with metastases to the lungs, bone, or bone marrow at diagnosis [1]. Owing to multicenter clinical trials, the survival for patients with ES, especially patients with localized disease has improved over the past decades with the application of systemic chemotherapy in conjunction with either surgery or radiation therapy or both for local tumor control. Currently, the 5-year overall survival in patients with localized ES is approximately 70%; however, this rate has plateaued over the past ten years. The prognosis of children and young adults with metastatic or recurrent disease is grim with less than one-third of patients with metastases at diagnosis and only 10% of patients with recurrent disease being long-term survivors [2, 3].

Unfortunately, the lack of survival gains over the last ten years for these high risk patients' groups suggests that further improvements in outcome with classic chemotherapy maybe limited. New targeted antineoplastic agents based on detailed insights into the biology of ES are needed.

The pathognomonic genetic marker of ES is the recurrent translocation involving the *EWS* locus on chromosome 22 band q12. In the majority of cases (85% to 90%), the amino terminus of *EWS* is juxtaposed with the carboxy terminus of *FLI1*, a member of the ETS family of transcription factors which is coded by a gene located on chromosome 11 band q24 [4]. To a lesser extent, other *ETS* family members that combine with the *EWS* gene include *ERG* (chromosome 21), *ETV1* (chromosome 7), and *E1AF* (chromosome 17) [5, 6]. The fusion of *EWS* with an *ETS* family member results in an aberrant transcription factor, altering cellular functions, and signalling pathways leading to improved survival, loss of differentiation, and proliferation. The cell first expressing the fusion transcript and responsible for tumor formation is a matter of debate, however, gene expression analysis suggests mesenchymal stem cells to be the cell of origin of ES [7]. Besides the translocation involving the *EWS* gene, additional numerical and structural aberrations have been observed in ES, including whole as well as partial chromosomal gains and losses [8]. These latter genetic changes will not be further discussed in this paper. We will focus on the effects of *EWS-FLI1* on apoptotic- and survival pathways and possible therapeutic targets. 

## 2. EWS-FLI1

**Figure 1:**
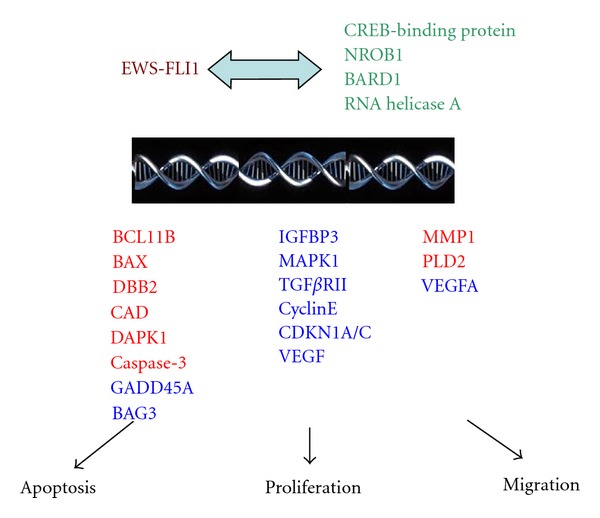
Interaction of *EWS-FLI1* with transcription factors (green). Examples of induced target genes are red, suppressed genes are blue. Alteration of gene expression by *EWS-FLI1* leads to suppression of apoptosis and enhancement of proliferation and migration.

The t(11; 22)(q24; q12) chromosomal translocation that fuses the *EWS* gene to the *FLI1* gene was first identified almost 20 years ago. The precise cellular mechanism by which *EWS*-*FLI1 *leads to ES still remains to be determined. The fusion protein product of *EWS-FLI1* preferentially binds to consensus *ETS* motifs and GGAA repeat microsatellite sequences. These binding sites are outside of the promoter regions and located up to more than 5 kb upstream of the regulated genes [9]. *EWS-FLI1* lacks a stable structure and contains a high proportion of disordered regions which facilitates interaction with a number of transcription factors as well as ease binding and dissociation from nuclear protein complexes to alter cellular transcriptional activities [10]. Moreover, direct protein interaction has been observed between *EWS-FLI1* and RNA polymerase II, CREB-binding protein, BARD1, NROB1 and RNA Helicase A (RHA) [10–12]. The sum effect of binding of *EWS-FLI1* to cellular components is threefold: (1) induction of transcription of genes involved in cell cycle regulation and DNA repair, (2) repression of expression of genes involved in cell adhesion, migration and homing such as integrin-, polysaccharide-, and glycosaminoglycan- or heparin-binding proteins, and (3) altered expression of several apoptotic genes (Figure 1) [11–13].

Because *EWS-FLI1* lacks enzymatic activity, recent research in ES therapy, has targeted the disruption of protein-protein interactions of *EWS-FLI1* and its binding partners. Erkizan et al. demonstrated that direct interaction between *EWS-FLI1* and RHA, was crucial for *EWS-FLI1*- induced transformation of mouse embryonic fibroblasts. After screening 3,000 small molecules from the NCI Drug Targeting Program, one small molecule, YK-4-279, which inhibited the interaction between *EWS-FLI1* and RHA was identified. YK-4-279 induced the activation of caspase-3 and apoptosis in ES cell lines and inhibited tumor growth in an ES xenograft model but not in malignant non-*EWS-FLI1*-expressing cells [14]. Another modulator of *EWS-FLI1* activity, however, did not prove to be effective in ES patients. Cytarabine, an antimetabolite antineoplastic agent, reduced EWS-FLI protein abundance in ES cells, diminished cell viability *in vitro* and abrogated tumor growth in a xenograft model by inducing apoptosis and inhibiting anchorage independent growth [15]. In a phase II study, administration of intermediate dose cytarabine to ten patients failed to show any antitumor activity. Another trial did not find any benefit for the three ES patients treated with low dose cytarabine [16].

## 3. EWS-FLI1, TP53, RB Signalling Pathway, and CDKs

Early investigations of *EWS-FLI1*-induced transformation of mouse embryonic fibroblasts (MEFs) revealed that not all fibroblasts were equally susceptible to transformation. Overexpression of *EWS-FLI1* in normal MEF resulted in apoptosis and growth arrest [17, 18]. In contrast, transformable MEFs lacked components of the G1 checkpoint, that is, p16^ink4^ or tp53, favoring stable expression of *EWS-FLI1* to hinder apoptosis. When injected with *EWS-FLI1*-transduced MEF, only two of eight SCID mice developed tumors, suggesting that further cooperating genetic events are required for tumorigenesis in this experimental system [18]. 

In human neonatal foreskin fibroblasts growth arrest induced by stable expression of *EWS-FLI1* was a result of TP53-upregulation and independent of alterations of the p16-RB pathway. Inhibition of TP53 bypassed an early growth arrest. Nevertheless additional *EWS-FLI1*-induced growth inhibitory pathways have to be suppressed in order to transform human fibroblasts. Even in transformed cells expressing E6, E7, SV40, and RAS^V12^ induction of *EWS-FLI1* inhibits colony formation. The characterization of these additional inhibitory pathways induced by *EWS-FLI1* is pending. The independence of *EWS-FLI1*-induced growth arrest from p16 pathways in this cellular background emphasizes the importance of careful interpretation of results in different cellular model systems [19]. By contrast, expression of *EWS-FLI* in murine bone-marrow-derived mesenchymal progenitor cells in the presence of functional tp53 generated tumors that displayed hallmarks of ES, providing a clue to the potential origin of ES [7, 17]. The basis for the susceptibility of these mesenchymal cells to transfection is currently focus of intensive research.

New data show direct interaction of *EWS-FLI1* N-Terminal Domain with TP53 protein in the nucleus and binding of this complex to the promotors of *P21* and *MDM2*, inhibiting their expression [20]. Another study using *TP53* wt ES cell lines showed that disruption of NOTCH signalling by *EWS-FLI1* through repression of JAG1 led to repression of TP53 and downstream P21-mediated cell cycle arrest. In addition, inhibition of the NOTCH signalling pathway by *EWS-FLI1* might suppress terminal differentiation in the ES precursor cell which could promote transformation and tumor growth [21].

While mutations or deletions of a cell cycle checkpoint gene such as *TP53* and *CDKN2A* are commonly encountered in many tumor types, in ES, specific mutation or deletion of these genes are rare events [22]. For example, most ES have TP53 wt and only 5% to 20% harbor deletions or point mutations of TP53, 30% show deletions of  *p*16^INK4^ [18, 23]. Aberrations of TP53 or p16^INK4^/p14ARF, although rare, are associated with decreased overall survival and have been the strongest negative predictor of outcome in ES, even more than the presence of metastases at diagnosis in multivariate analysis [22]. The apparent contradiction between rare TP53 mutations and data suggesting a need of inhibition of the TP53 pathway to transform the cell of origin is yet not solved but could serve as a clue to understand the initial steps in development of ES.

In addition to TP53 inhibition, *EWS-FLI1* induces overexpression of CDKs in ES cells. Since inhibition of CDKs has been shown to trigger apoptosis via the intrinsic pathway in various tumor models, CDK may present a potential target for therapy in ES [12]. Tirado et al. found that roscovitine, a purine analog and potent CDK inhibitor, was a highly efficient inducer of apoptosis in TP53mut ES cells via TP53-independent upregulation of the proapoptotic protein BAX and downregulation of survivin and XIAP leading to caspase-3 and caspase-7 activation [24]. Likewise, Li et al. treated WE-68, a *TP53*wt ES cell line, with flavopiridol, a pan CDK inhibitor which induced TP53, resulting in an increase of BAX/BCL-2 ratio and the release of mitochondrial cytochrome c and the activation of caspase-9, caspase-8, and caspase-3 and apoptotic cell death [25]. 

## 4. BCL-2, IAPs, and Smac/DIABLO

Based on the observation that expression of *EWS-FLI1* in nontransformed primary cells results in apoptosis and that ES cell lines are exquisitely chemosensitive, changes in ratios of pro- and antiapoptotic members like BAX/BAK, BH3-only proteins (i.e., BID, BAD, Puma, NOXA) and BCL-2/BCL-X_L_ in ES may be supposed. Gene expression analysis showed that although the majority of genes regulated by *EWS-FLI1* belong to cell cycle control and differentiation pathways, some apoptotic genes like BCL11B (antiapoptotic-induced), GADD45A (proapoptotic-repressed), CAD (execution of apoptosis–induced), DAPK1 (proapoptotic-induced), BAG3 (antiapoptotic-repressed), DBB2 (proapoptotic-induced), and Caspase-3 (proapoptotic-induced) are also regulated. Detailed functional studies of most of these factors in ES cell lines are lacking [12]. Similar to the above-mentioned analysis of cell cycle control in different cellular model systems, the *EWS-FLI1* expression pattern and its effects on apoptosis are likely to be cell type-dependent, as well. In cellular model systems which are only partially comparable to the cell of origin, the accumulation of proapoptotic factors like caspase-3 leads to initiation of apoptosis in case of *EWS-FLI1* expression [26]. This intriguingly raises the question why the mesenchymal stem cell is resistant to the apoptotic stimuli mediated by *EWS-FLI1* expression: the answer may help to identify new therapeutic targets. IAP (inhibitors of apoptosis) such as Smac/DIABLO in ES have garnered less attention. There has been only one study, analysing primary tumour tissues, which reports the expression of Smac/DIABLO in the cytoplasm of one of two primary ES tumor samples [27].

## 5. Death Ligands and Receptors

**Figure 2:**
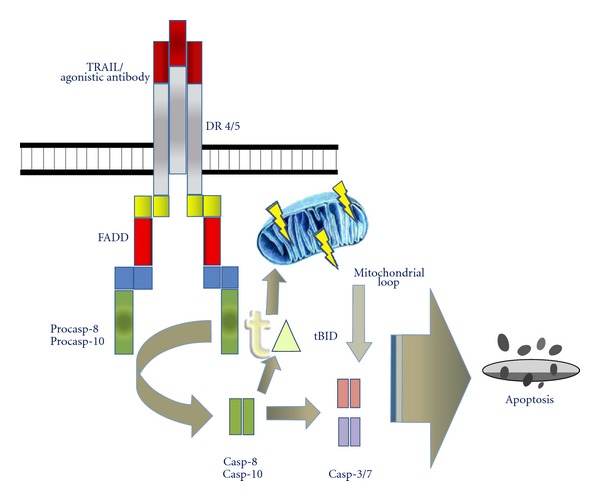
Initiation of apoptosis and death signalling through TRAIL receptors.

Apoptosis via death receptors serves as the principal pathway in immune-mediated antitumour response and represents an attractive target for therapy. In susceptible cells, interaction of a death ligand with its corresponding death receptor leads to formation of DISC (death-induced signalling complex) and direct high level of activation of caspase-8 and downstream effector caspases (type I cells). In cells with only low levels of activated caspase-8 generated (type II cells), activation of downstream caspases is mediated via the mitochondrial loop after cleavage of BID, a BH3 domain containing BCL-2 family protein, by caspase-8 [28]. FAS-FASL-, and DR4-/DR5-TRAIL- (TNF-related apoptosis-inducing ligand)-induced apoptosis represent prototypical apoptotic signalling pathways (Figure 2) [29]. Although FAS and FASL are expressed in a wide range of ES cell lines, most ES cell lines are not sensitive to FAS-induced apoptosis. Additional inhibition of antiapoptotic factors by cycloheximide or upregulation of proapoptotic factors such as by IFN-*γ* are often required for FAS-mediated apoptosis in ES cells [30]. Deregulation of BCL2 family members may be responsible for this resistance as FAS-sensitive cell lines had a higher expression of the proapoptotic protein BAD and lower levels of the antiapoptotic protein BAR [31]. In contrast to the inconsistent sensitivity to FAS-mediated apoptosis in ES cells, TRAIL induces apoptosis in about 80% of ES cell lines expressing the corresponding DR4 and DR5 death receptors *in vitro *[32, 33]. Nevertheless, a fraction of ES cells is resistant to TRAIL-induced apoptosis by virtue of low or absent expression of caspase-8 and subsequent inhibition of the downstream apoptotic cascade [34]. Interestingly, caspase-8 may be upregulated by interferon-*γ* (IFN-*γ*) via the STAT1 pathway, to render previously resistant cells susceptible to TRAIL [3, 35]. Concentrations of IFN-*γ* required for TRAIL induced apoptosis in these ES cells were as low as 20 U/mL; an amount fourfold lower than concentrations found in sera of patients treated with IFN-*γ* for other diseases [34].

Efforts to demonstrate an antitumor effect of TRAIL *in vivo*, however, have not been successful to date. In an orthotopic ES mouse xenograft model, TRAIL demonstrated only moderate antitumor activity in primary tumors, but had no effect on metastases. Combined therapy with doxorubicin, which has been shown to induce DR4 and DR5 in renal cell carcinoma cells, did not improve efficacy. Mechanisms involved in TRAIL resistance in this model are yet not clear. In addition to other changes in TRAIL signalling, downregulation of DR4 may be important [36]. In another model, gene delivery utilizing a cationic lipid vector led to sustained expression of hTRAIL in tumor cells and inhibited growth of the primary tumor and improved overall survival in mice. Unfortunately, metastases could not be evaluated due to a low rate of metastatic lesions [37].

To overcome resistance of tumor cells to TRAIL-mediated apoptosis, an increasing number of combinational therapies have been investigated both *in vitro* and *in vivo* studies. Treatment with IFN-*γ* and TRAIL-receptor antibodies decreased metastasis formation and improved overall survival in mice, although only a modest effect on primary tumor growth was observed [36]. Mechanisms of how and why IFN-*γ* prevented metastasis are under investigation. Another therapy showing synergistic effect in two ES cell lines was treatment with histone deacetylase inhibitors and TRAIL [38]. Similarly, application of TRAIL and the proteasome inhibitor bortezomib to ES cell lines resulted in cell cycle arrest and apoptosis [39]. In a mouse model, combination treatment with TRAIL and the tyrosine kinase inhibitor imatinib reduced both the volume of primary tumours as well as pulmonary metastases possibly due to imatinib-induced enhancement of NK-cell sensitivity to IL-2 or IL-12 leading to increased IFN-*γ* release and stimulation of TRAIL-downstream pathways. Notably, in contrast to previous studies, treatment with TRAIL alone reduced development of pulmonary metastases in this model [40, 41].

Use of TRAIL as a single agent in phase I and II trials in humans showed that the agent was well tolerated but had limited efficacy. In three phase I studies in solid tumors, monotherapy with human monoclonal antibodies against TRAIL receptor, demonstrated stable disease at best [42–44]. Considering that any oncologic monotherapy leads to rapid development of therapeutic resistance, combinational therapeutic approaches have been designed in order to interfere with TRAIL resistance through modulation of the TRAIL-signalling pathway. A phase Ib study combining recombinant hTRAIL with paclitaxel, carboplatin, and the antivascular endothelial growth factor A (VEGF-A) agent bevacizumab, in patients with advanced NSCLC showed that combination therapy was well tolerated with no dose limiting toxicities. The authors were able to demonstrate an antitumor activity with a 58% overall response rate [45]. Of note, variations in the expression of DR4 and DR5 or caspase-8 in different tumor types, different patients and perhaps different cells within one tumor have been observed. These potential limitations of death ligands as antitumor agents highlight the importance of combination regimens when using death receptors as therapeutic targets [34].

## 6. The IGF-I/IGF-IR Signalling Pathway

**Figure 3:**
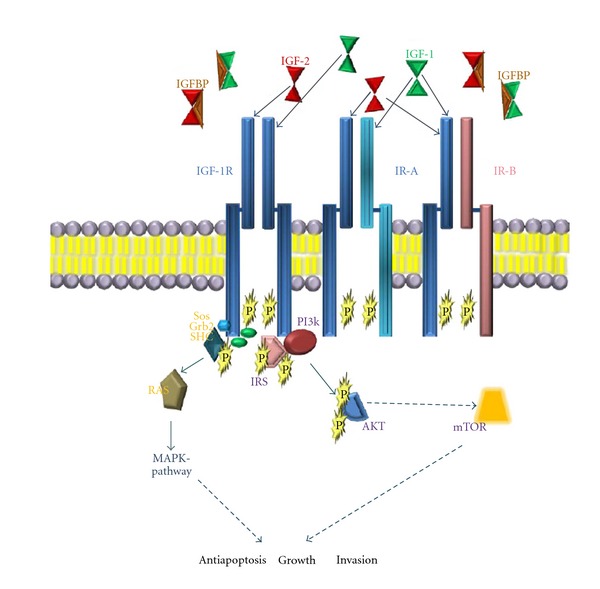
Insulin receptor, ligands and inhibitory components leading to activation of the MAPK- and PI3K- pathway. Solid lines indicate direct interaction of participating factors, broken lines indicate final effects (i.e. activation of mTOR).

The contribution of the insulin-like growth factor (IGF) pathway to oncogenesis in ES is widely accepted [46, 47]. Binding of IGF-I to the insulin-like growth factor I receptor (IGF-IR) leads to activation of PI3K- and MAPK-pathways promoting proliferation (Figure 3) [48]. Several epidemiological studies suggest a link between IGF-I and ES. The peak incidence of ES in the second decade of life and rising IGF-I levels in puberty appear to be a fundamental part of tumour initiation rather than mere co-incidence [1, 49]. Patients with metastatic disease and low IGF-I levels and high IGFBP3:IGF-I ratios showed a trend towards improved survival [50]. Moreover, there is data linking elevated IGF-I blood levels to increased risk of breast, colon or prostate cancer [48, 51]. Molecular studies have shown that (1) the aberrant transcription driven by *EWS-FLI1* leads to repression of insulin-like growth factor binding protein 3 (IGFBP3) [52], (2) IGF-I is induced by the ES fusion protein [53] and (3) in NIH3T3 cells and embryonic stem cells, in addition to direct IGFBP3 repression, IGFBP3 expression is further diminished by EWS-ETS repression of transforming growth factor beta receptor type II (TGF*β*RII) expression. TGF*β* was shown to induce IGFBP3 and to mediate growth inhibition in breast cancer cells [54–56].

There are preclinical and clinical studies examining the therapeutic effects of targeted therapy to IGF-IR or downstream components of the pathway. Induction of apoptosis through inhibition of IGF-IR with a monoclonal antibody was observed as early as 1990 [57–59]. Combination therapy with classic chemotherapy agents increased apoptosis and impaired the formation of colonies in soft agar [60]. Based on these encouraging results, several IGF-IR antibodies have been developed and are being tested in phase I/II studies in ES patients. Treatment with figitumumab, an IgG2 anti-IGF-IR-monoclonal antibody, resulted in one complete and one partial response in a cohort of 15 ES patients. Forty percent (*n* = 6) of patients had stable disease lasting from 4 to >16 months. Six patients were free of disease progression after 6 months of IGF-IR blockade [61]. Similar results were achieved in ES patients with another IgG1 anti-IGF-IR-antibody, R1507. Two of nine (22%) patients had a partial response while another two patients had stable disease [62]. Although response rates are only around 25%, these results are promising because the responses were observed with administration of one single-agent in the setting of recurrent disease in patients who had previously received multiple chemotherapy courses. Indeed, future studies may benefit from molecular studies to identify patients most likely to respond to IGF-IR-antibody therapy [63].

Other therapeutic strategies have used tyrosine kinase inhibitors targeting members of the IGF-IR signaling pathway. *In vitro* data on IGF-IR kinase inhibitors such as NVP-AEW541, ADW742, and GSK1904529A showed induction of apoptosis and G1 arrest in ES cell lines. Combination therapy with vincristine or doxorubicin led to additive effects [64–66]. NVP-AEW541 and GSK1904529A also showed antitumor activity in xenograft tumors in mice [66, 67]. Similar results including apoptosis, G1 arrest, and inhibition of cell migration were observed with the tyrosine kinase signaling inhibitors, PD98059 and U0126, which inhibit MEK/MAPK, and LY294002, which inhibits PI3K when used in combination with chemotherapeutic drugs *in vitro* [68].

Silencing *EWS-FLI1*, which leads to inhibition of IGF-IR expression, in combination with inhibitors of downstream kinases, NVP-AEW541, LY294002, and PD98059, had synergistic effect on apoptosis in one ES cell line raising the question whether direct IGF-IR blockade should be combined with inhibition of downstream pathway players to increase therapeutic responses [69]. mTOR which serves as a target of many of tyrosine kinase pathways is of special interest in this regard. Hyperphosphorylation of mTOR and other downstream IGF-IR mediators like ERK and AKT defines an unfavorable subgroup of ES patients [70]. Combined inhibition of IGF-IR and mTOR by cixutumumab, a humanized anti-IGF-IR-IgG1 monoclonal antibody and temsirolimus led to a >20% tumor volume reduction in two of three ES patients. An ongoing phase II clinical trial will determine whether these results may be verified in an expanded cohort of ES patients [71].

## 7. Tyrosine Kinases as Targets

The groundbreaking results of tyrosine kinase inhibitor imatinib in the treatment of patients with chronic myelogenous leukemia (CML) and gastrointestinal stroma tumors (GISTs) represent a perfect example of translating basic research into development of new drugs. Thusly, the expression and role of kinases have been studied in ES [72–75].

C-kit may be detected in 38–44% of primary ES tumors. Cell lines expressing the receptor showed growth inhibition ranging between 20 to 40% without significant apoptosis* in vitro* at clinically relevant doses of imatinib. Combination treatment with doxorubicin and vincristine had synergistic effects with 15 to 30% apoptosis compared to controls [76, 77]. The therapeutic value of imatinib was studied in a phase II clinical trial by the Children's Oncology Group and unfortunately had disappointing results. Imatinib efficacy in 24 patients with ES was low with only one partial response observed [78, 79]. Similar to imatinib, dasatinib, a broad spectrum tyrosine kinase inhibitor, which induces apoptosis in ES cell lines *in vitro*, was not able to show any therapeutic efficacy in ES patients [80, 81]. Recently, ABT-869, a tyrosine kinase inhibitor targeting Fms-like tyrosine kinase-3, c-kit, VEGF-Rs and PDGF-Rs, has been shown to reduce metastasis and spontaneous growth of ES xenografts in mice [82, 83].

A receptor tyrosine kinase well characterized in breast cancer is HER-2/*neu*. The receptor belongs to the EGF-receptor family and activation is associated with promotion of cell growth, inhibition of differentiation and apoptosis via activation of PI3K, MAPK, and STAT pathways [84]. HER-2/*neu* overexpression is found in a variety of ES cell lines and in 16% of primary tumors; overexpression, however, does not correlate with prognosis [85, 86]. Treatment of ES cells with trastuzumab, a monoclonal antibody targeting HER-2/*neu*, inhibited cell growth *in vitro*. Combined treatment with taxol but not with etoposide, doxorubicin or 9-nitrocamptothecin had a synergistic effect on growth inhibition *in vitro* and *in vivo *[86]. However, tumor growth in mice was only delayed suggesting that trastuzumab has modest clinical effect in ES.

Resistance to tyrosine kinase inhibition is a common event even in a malignancy like CML, which is very sensitive to specific inhibitors [87]. Combinational inhibition of different tyrosine kinases, which substitute for each in case of primary and secondary resistance, might be an opportunity to hinder disease progression and to improve overall survival. Huang et al. showed that primary resistance to the IGF-IR inhibitor, BMS-536924, was due to overexpression of EGFR in one ES cell line. In a resistant rhabdomyosarcoma cell line, combination of BMS-536924, with the pan-HER-2 inhibitor, gefitinib, had synergistic antiproliferative and apoptotic effects [88]. The combination of different tyrosine kinase inhibitors could serve as a platform for future designs of clinical trials. Moreover, combination of tyrosine kinase inhibitors with chemotherapeutic drugs may confer additive antitumor activity.

## 8. Conclusion

Expression of *EWS-FLI1* in the cell of origin of ES likely represents the decisive transformational event initiating prosurvival, proproliferation and prometastatic pathways which ultimately results in clinically apparent ES. Characterization of these pathways will identify new therapeutic targets which are required to improve survival particularly for ES patients with metastatic and recurrent disease. This goal will most likely be achieved by combinational inhibition of specific prosurvival pathways in conjunction with induction of apoptosis through DNA damage induced by current classic chemotherapeutic drugs as well as targeted agents, such as death ligands, tyrosine kinase inhibitors, and/or anti-IGF-IR-antibodies.
